# Disorders of the Oesophagus -Advances and Controversies

**Published:** 1985-07

**Authors:** 


### DISORDERS OF THE OESOPHAGUS – ADVANCES AND CONTROVERSIES

**Edited by A. Watson and L. R. Celestin**
**Pitman. Price £27.95**

This book is the result of a Symposium held at Lancaster in 1984 and organised by the compilers. About half the invited speakers were from abroad, including five from the U.S.A. with others from Belgium, France, Holland, Italy, Canada and South Africa, all acknowledged experts in the field of Oesophageal disorder. The papers were presented in four sections – Investigation/Motor Disorders, Gastro-oesophageal reflux, Carcinoma of the Oesophagus and Oesophageal Varices/Paediatric problems. Bristol contributors were the joint editor Mr. L. R. Celestin and Miss H. R. Noblett. Each section was concluded with a Chairman's Overview in which he gave his own thoughts on the papers by the various speakers. The result is a comprehensive survey of present knowledge on oesophageal disorders and current trends in treatment. The melancholy results of surgery for both oesophageal carcinoma and oesophageal varices emphasise the need for understanding aetiology and helping prevention and these aspects are given prominence in the symposium. Both conditions are alcohol related and the former is also related to smoking and narcotic abuse as well as to nutritional deficiency. An interesting league table of incidence of oesophageal carcinoma put France at the head for men and Scotland for women, though figures were not available for some third world countries known to have a high incidence. The surprising fact emerged that Lancaster is an enclave with an above average incidence of oesophageal carcinoma, and that this fact was not unconnected with the holding of the conference there. Mr. Watson of Lancaster and Mr. Celestin are to be congratuated on organising this symposium and for producing this extremely useful account of its proceedings. It will be of great interest and use to all gastro-enterologists.

